# Physical Activity and Quality of Life of Healthy Children and Patients with Hematological Cancers

**DOI:** 10.3390/ijerph16152776

**Published:** 2019-08-03

**Authors:** Aleksandra Kowaluk, Marek Woźniewski, Iwona Malicka

**Affiliations:** Department of Rehabilitation in Internal Medicine, Faculty of Physiotherapy, University School of Physical Education, 51-612 Wroclaw, Poland

**Keywords:** physical activity level, quality of life, child health, cancer

## Abstract

The aim was to assess the level of physical activity and the quality of life of children undergoing cancer treatment, during and after the completion of the treatment. Eighty-eight children aged 11–15 were enrolled. Three groups of children were assessed, i.e., children undergoing cancer treatment (*n* = 30), children after cancer treatment (*n* = 28), and healthy children (*n* = 30). The level of physical activity in children was assessed using the questions from the Health Behavior in School-Aged Children (HBSC) questionnaire. The assessment of children’s quality of life was conducted using the KIDSCREEN-10 Index. The chi-square test was used to assess the statistical significance of the differences in the results between the study groups in the case of both HBSC and KIDSCREEN-10 questionnaires. Children undergoing cancer treatment did not perform any physical activity of at least 60 min (in total) per day, during the week. Therefore, they did not meet the recommendations related to the appropriate level of daily physical activity (Moderate-to-Vigorous Physical Activity; MVPA). Children after cancer treatment and healthy children significantly more frequently undertook physical activity. The quality of life of children with cancer is significantly lower and different from the quality of life of healthy children.

## 1. Introduction

The increasing effectiveness of treatment in pediatric oncology results in the fact that more children are successfully cured or achieve a long-term remission [1]. In light of the above information, future health and the quality of life of these children become more significant. Treatment of childhood cancer is long-lasting and aggressive. Frequent hospitalizations, invasive examinations, lack of contact with peers, and subordination of life to the disease [2,3] lead to a decreased quality of life [4] and a significant reduction in the level of physical activity [5]. The prolonged periods of inactivity contribute to the reduced capacity of the cardiovascular system, reduced bone mineral density and muscle strength, and deterioration of physical fitness [6,7,8]. Decreased ability to perform daily activities [9] adversely affects the well-being and significantly reduces the quality of life of children undergoing treatment [4]. As a result, the dynamic development of the child’s psychological and social spheres during the treatment and at the time of recovery is disturbed [10,11].

Physical activity in children was previously an underrated part of the cancer treatment process. Currently, this approach has changed and physical activity is promoted at every stage of the treatment [12,13,14]. Studies revealed that physical activity in a child undergoing cancer treatment prevents many functional deficits and shortens the convalescence time [15]. Additionally, it has a beneficial effect on the specific immune response in children, after a bone marrow transplantation [16].

Due to their health conditions, children undergoing cancer are reluctant to undertake physical activity, which is an essential part of the child’s balanced development [17]. There are many guidelines on the forms of physical activity for adult cancer patients, which are clearly defined [18]. In the group of children with cancer, no uniform recommendations have been developed yet. Studies showed a positive effect of physical exercise on the physical and psychosocial spheres in children. Adverse effects related to such activities have not been observed. Children undergoing cancer treatment should also undertake physical activity and should not be deprived of it [12,13,14,15,16].

It was revealed that the physical activity of children after cancer is still reduced and it is not only due to treatment-related adverse effects but also due to bad habits acquired during cancer and the overly cautious attitude of parents and educators [19]. An adequate level of physical activity in childhood determines the fitness and health of adults. It is also a preventive factor for the diseases of affluence.

Promotion of physical activity in children during and after cancer treatment is of great importance. The physical activity in such patients is not contraindicated. Quite the contrary, it should be promoted. Adjusting the type and intensity of exercise to individual abilities of each child and to the stage of cancer treatment is also of crucial significance [12,20,21]. Carefully selected exercise programs can help alleviate the adverse effects of cancer treatment, minimize functional deficits, and significantly reduce the convalescence time [15].

The present recommendations on the level of physical activity are mainly related to healthy children. The recommended intensity of physical activity that is necessary for the proper development of a child should be at least at the moderate level, minimum 5 days per week, each lasting at least 1 h [22,23]. Studies confirm a beneficial effect of moderate to vigorous physical activity (MVPA) on health indicators in children and adolescents [22,24]. However, they are usually related to meeting the recommendations on the appropriate level of MVPA in the group of healthy children [25]. The importance of MVPA should not be overestimated. Some reports indicate that physical activity of light intensity has a positive effect on children’s health indicators [26]. Activity in any form is important to children.

The aim of the study was to assess the level of physical activity and the quality of life of children during and after cancer treatment. This assessment might help to develop rehabilitation programs and to determine an individual’s level of physical activity.

## 2. Materials and Methods

### 2.1. Study Group

Eighty-eight children aged 11–15 were enrolled in the study (Table 1). Three groups of children were examined, i.e., children undergoing cancer treatment, children after cancer treatment, and healthy children. The groups were selected in such a manner as to show to what extent cancer and its treatment influenced the daily activity and the quality of life of children. The selection of such groups also allowed to determine to what extent these parameters were different from the results of healthy children. Additionally, it allowed us to assess whether past cancer treatment affected the physical and psychosocial spheres in children. The inclusion and exclusion criteria were defined (Table 2).

Group I consisted of the patients of the Department of Pediatric Bone Marrow Transplantation, Oncology and Hematology, University Teaching Hospital, Wrocław, Poland. Group II was comprised of children who were the participants of the Lower Silesian Onco-Olympic Games of Children and Adolescents (sports competition aimed at promoting physical activity among children treated for cancer). Group III consisted of junior high school students with a negative history of cancer.

The groups were comparable in terms of the number of subjects, age, gender, weight, and height (Table 1). Group I consisted of 30 children, i.e., 13 girls and 17 boys who were hospitalized due to acute lymphoblastic leukemia (*n* = 24) and acute myeloid leukemia (*n* = 6). Chemotherapy was the form of treatment in Group I (mean treatment duration 2.2 years). Group II consisted of 28 children after cancer treatment (girls *n* = 17, boys *n* = 11). These subjects had been previously diagnosed with acute lymphoblastic leukemia (*n* = 16), acute myeloid leukemia (*n* = 4), Ewing’s sarcoma (*n* = 3), and Hodgkin’s lymphoma (*n* = 5). Chemotherapy was the basic form of treatment in Group II. The time from treatment completion in children from Group II was > 1 year. Group III (*n* = 30) included healthy children with no history of cancer or other chronic diseases (girls *n* = 14, boys *n* = 16).

### 2.2. Research Methods

The level of physical activity of the subjects was assessed using the questions from the Health Behavior in School-Aged Children (HBSC) questionnaire from the section related to health behavior. The questions were connected with physical activity within the last seven days. We assessed the number of days in a week during which children exercised for at least 60 min—MVPA. The task of the subjects was to estimate the total daily amount of time they spent on physical activity. The frequency and duration of significant physical effort were also assessed. The effort was defined as any activity that resulted in an increased heart rate, temporary shortness of breath, and increased sweating. The questionnaire also included three questions related to sedentary behavior and the time spent in a sitting position in front of a TV or a computer screen.

The assessment of the quality of life of the subjects was conducted using the KIDSCREEN-10 (the short health-related quality of life questionnaire). The short version of the questionnaire assesses the health-related quality of life of children and adolescents. The answers are given based on the last seven days. Subjective feelings of the children related to the condition of their physical and mental health were assessed on the basis of the first four questions of the questionnaire. The relationships with parents and peers, the child’s autonomy, and feelings related to the school environment were also assessed.

In each study group, an anonymous questionnaire survey was conducted in the traditional paper form.

### 2.3. Ethics

The study was approved by the Local Bioethics Committee at the University of Physical Education in Wroclaw, Poland (consent no 22/2018).

### 2.4. Statistical Analysis

Statistical analysis was performed in GraphPad Prism 7 (Institute of Immunology and Experimental Therapy, Wroclaw, Poland). The normality of the data distribution was assessed using the Shapiro-Wilk test. Parameters defining the characteristics of the study groups were presented by providing the descriptive statistics, such as arithmetic mean, median, and lower and upper quartile. The chi-square test was used to assess the statistical significance of the differences in the results between the study groups in the case of HBSC and the KIDSCREEN-10 questionnaires. The level of significance was set at *p* < 0.05.

## 3. Results

### 3.1. Physical Activity Level

Children undergoing treatment did not perform any physical activity during a week that lasted at least 60 min in total daily and, therefore, they did not meet the recommendations related to the appropriate level of daily physical activity (MVPA). After completing cancer treatment, the subjects declared that they were physically active at least once a week for a minimum period of 60 min per day, compared to children undergoing treatment. Healthy children (40%) undertook such physical activity at least 5 days per week (Table 3).

In the majority of cases, children undergoing cancer treatment did not undertake significant physical activity that led to general fatigue. After completing the cancer treatment, the subjects spent at least 30 min per week doing vigorous physical activity. Eighty percent of children undergoing cancer treatment did not undertake vigorous physical activity. Thirty six percent of children undertook vigorous physical activity even 4–6 times per week, which lasted at least 2 h per week (Table 3).

Almost 90% of children undergoing cancer treatment spent at least two hours per day in front of a TV screen. Children after treatment completion and healthy subjects spent significantly less time during the day in front of a TV screen. A comparable number of the subjects spent more than 2 h per day watching TV (67.8% of children from Group II and 60.0% from Group III; Table 4).

The whole group of children undergoing cancer treatment used a computer for 30–60 min per day. Almost 70% of children from Group I reported that they also played games in addition to performing other activities that required the use of a computer. The subjects from Groups II and III spent significantly more time in front of a computer screen. The vast majority of children from Groups II and III spent at least one hour per day in front of a computer screen (82.1% and 86.6%, respectively; Table 4).

On off-days, children from Group I spent more time in front of a TV screen (at least 2 h per day) compared to other days of the week. Almost 68% of the subjects from Group II and 73% of the subjects from Group III spent at least 2 h per day in front of a television screen on off-days. The amount of time devoted to playing games on off-days increased in each group compared to other days of the week. The percentage of the children who played games at least 30 min per day was as follows—86.7% from Group I, 78.5% from Group II, and 83.3% from Group III (Table 5).

### 3.2. Quality of Life

All of the subjects who were undergoing treatment for cancer did not feel fit or well. The children who completed cancer treatment assessed their well-being and physical fitness significantly better. Almost 36% of the subjects from Group II reported at least very good well-being and physical fitness, whereas 83.3% of the children from Group III assessed their health as at least very good. All children undergoing cancer treatment reported the lack of energy and fatigue and 86% of the subjects from Group II reported at least a frequent feeling of energy and willingness to undertake the physical activity. Seventy percent of the healthy children reported an increased energy level and willingness to undertake physical activity. All subjects undergoing cancer treatment experienced sadness within the last 7 days. The subjects who completed the treatment and the healthy subjects reported the absence of low mood within the last 7 days (35.7% and 30%, respectively). The distribution of the answers of the subjects from Groups II and III to the question related to the mood and the feeling of sadness within the last seven days was similar. A comparable number of the subjects from each group rarely felt lonely (Table 6).

The level of autonomy of the subjects undergoing cancer treatment was significantly lower compared to the subjects from Groups II and III. As many as 43% of the subjects undergoing cancer treatment reported that they had an insufficient amount of time for themselves. The vast majority of the subjects from Groups II (64.3%) and III (66.7%) reported that they had time for themselves at least very often (Table 7).

The subjects from Groups I, II, and III reported very good relations with their parents. The subjects undergoing cancer treatment mostly reported an insufficient contact with their peers (Table 8).

As many as 90% of the subjects undergoing cancer treatment reported the lack of satisfactory results at school. Children after the completion of cancer treatment and healthy subjects reported significantly greater satisfaction with their school results. All subjects from Group I reported decreased concentration and attention-related problems. The subjects from Groups II and III reported good concentration (89.3% and 90%, respectively) (Table 9).

## 4. Discussion

Cancer treatment process is long-lasting and debilitating. It adversely affects the level of physical activity and the quality of life of pediatric patients and survivors.

Aggressive protocols for anticancer therapy lead to many adverse effects that are observed during treatment and many years after its completion. Sarcopenia is one of the most common problems resulting from the catabolic action of several chemotherapeutic agents. The occurrence of muscular atrophy results in reduced muscle strength and a significant decline in physical performance [27,28]. Limited exercise capacity enhances protective lifestyle and the cessation of daily physical activity [29].

Treatment duration of childhood cancer significantly reduces the frequency of performing even light physical activity and recreational sports, such as everyday walks and playing with peers in the open air. The level of physical activity of children with cancer also decreases significantly compared to the time before the disease. Reduced level of physical activity is observed in children suffering from cancer, during their stay at home and during hospitalization, which was found to be reduced by 74% to 91%, respectively [30].

During the cancer treatment, none of the subjects met the recommendations related to the appropriate level of physical activity per week. These criteria were met by 25% of the subjects after the completion of treatment, while they were met by 40% of the healthy children. Similar results were presented by Tan et al. Their subjects undergoing cancer treatment did not undertake the physical activity lasting a total of at least 60 min daily (MVPA) [5]. Additionally, Anzar et al. confirmed the decreased MVPA. In their study group, none of the children performed a 60-min physical effort for a minimum of five days per week [19].

The subjects, regardless of the group, preferred a sedentary lifestyle; all subjects also reported that they used a computer every day. In this case, however, both the subjects after cancer treatment and the healthy subjects spent significantly more time on this activity. It probably resulted from more activities related to learning and communication with peers. The amount of time in front of a computer/TV screen increased in each group during the days free of classes.

Lack of physical activity and the majority of time being spent in the sitting position in children with cancer, significantly reduced CRF. It is manifested by a lower peak oxygen uptake (VO2peak) in relation to the normal range (31.7 versus 45.1 mL/kg/min). Braam et al. showed that over 50% of children undergoing cancer treatment had a VO2peak below the normal value. Cardiorespiratory fitness is significantly associated with the level of physical activity and sedentary habits—any additional activity per minute results in an increase in VO2peak by 0.05 mL/kg/min, whereas each additional minute of sitting reduces VO2peak by 0.06 mL/kg/min [21].

A complete lack of physical activity among cancer children is a decisive factor that negatively affects the quality of life of these children and forms the belief about lack of independence [4]. Our study results showed that the children undergoing cancer treatment presented with a lack of well-being and decreased physical fitness. Children after cancer treatment had significantly better well-being and physical fitness.

Significant deterioration of physical and mental health, especially in the group of children who underwent chemotherapy and radiotherapy, was also confirmed by Bhat et al. Children manifested disorders related to emotional and social functioning [31].

In our studies, as many as 90% of the subjects undergoing cancer treatment reported learning difficulties and all of these subjects reported attention- and concentration-related problems. Both, the subjects after cancer treatment and the healthy children reported significantly greater satisfaction with their results at school, which is in line with observations of Bhat et al. Lack of contact with peers and the social environment might be the reason for social isolation and future communication problems [31].

All subjects undergoing cancer treatment were also characterized by excessive fatigue and a frequent lack of energy. Davies et al. reported that cancer treatment-related fatigue additionally enhances a protective lifestyle and the cessation of physical activity. Children manage their dwindling energy and minimized further loss of energy through preserving strategies, which include reduced physical activity and a sedentary lifestyle [32].

Physical activity is a factor that significantly determines the quality of life of children undergoing cancer treatment, which is confirmed by Speyer et al. and San Juan et al. Children who are physically active during hospitalization are characterized by a higher quality of life, and their self-esteem and activity-related satisfaction are improved [13,33].

Sadness is predominant in the subjects undergoing cancer treatment, compared to the other study groups. Moody et al. showed that a lack of contact with peers, health-related, and future-related anxiety and excessive concern of parents and of the medical personnel adversely affect satisfaction and the well-being of hospitalized children [34].

The effectiveness of treatment in pediatric oncology is increasing, and hence there is a higher number of survivors. Consequently, issues related to future health and the quality of life of these individuals become more significant. Individuals who were diagnosed in childhood with cancer are characterized by an increased risk of cardiovascular diseases [35], obesity [36], osteoporosis [37], and premature death [38]. Currently, physical activity is recommended at every stage of cancer treatment despite the fact that it remains an underappreciated component of cancer prevention and therapy [12,39]. Children with cancer who undertake physical activity during the treatment process are able to participate more actively in social and professional life, in future.

## 5. Limitations

Certain study limitations should be considered. Further studies are warranted to confirm the results of the present study. Such studies should be conducted on larger samples with longer follow-up periods. The results of other studies assessing the level of physical activity of children undergoing treatment for cancer are in line with the results of our study [19,30]. The questionnaire methods are easily accessible and are quick diagnostic tools. However, the use of objective methods to assess the level of physical activity, such as an accelerometer, would practically allow the assessment of the activity and sedentary behavior in children. Another limitation is also related to the fact that the subjects presented with different diagnoses, particularly in the group of children after the completion of cancer treatment.

## 6. Future Research Directions

Further studies should focus on developing and verifying the effectiveness of the training program in children undergoing cancer treatment. Such a program should be directed primarily at increasing the level of daily physical activity and counteracting the sedentary lifestyle in this group of patients. The different forms of physical activity should be personalized, diverse, and attractive and should meet the expectations of pediatric patients. Any form of physical activity is important, including low-intensity activity, such as games or plays.

Educational programs for children, parents, and health professionals should be created. An overly cautious approach of caregivers of cancer children might lead to a situation when the daily life of these patients is subordinated to the disease. As a result, these children lose their natural need for physical activity. Promotion of physical activity should occur at every stage during and after cancer treatment.

## 7. Conclusions

Cancer and its treatment significantly reduce the level of physical activity in children. The subjects undergoing treatment for cancer had a significantly lower level of physical activity compared to children who have already undergone cancer treatment and healthy children. The quality of life of children with cancer is significantly lower and is definitely different from the quality of life of healthy children. Our subjects with cancer experienced deterioration in both physical and mental health. The completion of cancer treatment resulted in increased physical activity and the quality of life. However, the values of these parameters were lower compared to the group of healthy children.

## Figures and Tables

**Table 1:** Characteristics of the study groups.

Parameter	Group I Children Undergoing Treatment	Group II Children after Treatment	Group III Healthy Children
Age [years]	13 ± 1.5	13 ± 1.9	13 ± 0.47
Weight [kg]	47 ± 13	51 ± 11	50 ± 10
Height [cm]	156 ± 13	159 ± 12	163 ± 8.7

**Table 2:** Inclusion and exclusion criteria in the study groups.

Group I	Group II	Group III
**Inclusion Criteria**		
- diagnosed cancer disease - hospital treatment - duration of hospital stay > 7 days - chemotherapy	- past history of cancer - completed cancer treatment > 1 year - past chemotherapy - no contraindications to physical activity confirmed by the medical certificate	- no history of cancer disease - no medical contraindications to participate in physical education classes in school
**Exclusion Criteria**		
- lack of informed consent from parents or the child - intellectual disability of the child	- lack of informed consent from parents or the child - intellectual disability of the child - practicing competitive sports	- lack of informed consent from parents or the child - intellectual disability of the child - practicing competitive sports

**Table 3:** Physical activity level in children. The percentage of the respondents providing a given answer [%].

Question/Variable	Answer	Group I	Group II	Group III
The number of days per week in which the child performed the physical activity of at least 60 min (MVPA)—HBSC 1	0 days	100.00	17.9	-
	1 day	-	10.7	-
	2 days	-	25.0	-
	3 days	-	21.4	20.0
	4 days	-	-	40.0
	5 days	-	-	20.0
	6 days	-	7.1	6.7
	7 days	-	17.9	13.3
*p*	* <0.0001	** <0.0001	*** <0.0001	**** <0.0001
Frequency of undertaking vigorous physical activity—HBSC 2	daily	-	14.3	16.7
	4–6 times/week	-	7.1	36.7
	2–3 times/week	-	28.6	33.3
	once a week	-	17.9	10.0
	once a month	-	10.7	-
	< once a week	23.3	7.1	3.3
	never	76.7	14.3	-
*p*	* <0.0001	** <0.0001	*** <0.0001	**** 0.026
The number of hours per week devoted to vigorous physical activity—HBSC 3	none	80.0	14.3	-
	about 30 min	20.0	21.3	-
	about 1 h	-	17.9	-
	about 2 h	-	17.9	10.0
	about 3 h	-	10.7	23.3
	about 4 h	-	3.6	36.7
	about 5 h	-	3.6	16.7
	about 6 h	-	-	3.3
	about 7 h or more	-	10.7	10.0
*p*	* <0.0001	** <0.0001	*** <0.0001	**** 0.0003

Chi square test: * all groups, ** Group I versus Group III, *** Group I versus Group II, **** Group II versus Group III.

**Table 4:** Time spent in front of a computer/TV screen per week. The percentage of respondents providing a given answer [%].

Question/Variable	Answer	Group I	Group II	Group III
The number of hours in front of a TV screen per week—HBSC 4.1	none	-	17.9	16.7
	about 30 min/day	-	14.3	6.7
	about 1 h/day	13.3	-	16.7
	about 2 h/day	40.0	25.0	36.5
	about 3 h/day	33.4	21.4	-
	about 4 h/day	13.3	10.7	6.7
	about 5 h/day	-	7.1	6.7
	about 6 h/day	-	-	6.7
	about 7 h or more/day	-	3.6	3.3
*p*	* 0.0101	** 0.0101	*** 0.0102	**** 0.0652
The number of hours spent playing games per week—HBSC 5.1	none	33.3	32.2	33.4
	about 30 min/day	10.0	10.7	3.3
	about 1 h/day	40.0	7.1	13.3
	about 2 h/day	16.7	10.7	13.3
	about 3 h/day	-	14.3	23.4
	about 4 h/day	-	7.1	10.0
	about 5 h/day	-	3.6	-
	about 6 h/day	-	3.6	-
	about 7 h or more/day	-	10.7	3.3
*p*	* 0.0324	** 0.0132	*** 0.0169	**** 0.6676
The number of hours spent in front of a computer screen per week—HBSC 6.1	none	-	17.9	6.7
	about 30 min/day	43.3	-	6.7
	about 1 h/day	56.7	32.1	13.3
	about 2 h/day	-	17.9	23.3
	about 3 h/day	-	14.3	10.0
	about 4 h/day	-	3.6	20.0
	about 5 h/day	-	3.6	10.0
	about 6 h/day	-	-	6.7
	about 7 h or more/day	-	10.6	3.3
*p*	* <0.0001	** <0.0001	*** <0.0001	**** 0.1050

Chi square test: * all groups, ** Group I versus Group III, *** Group I versus Group II, **** Group II versus Group III.

**Table 5:** Time spent in front of a computer/TV screen on off-days. The percentage of respondents providing a given answer [%].

Question/Variable	Answer	Group I	Group II	Group III
The number of hours in front of a TV screen at the weekend—HBSC 4.2	none	-	14.3	16.7
	about 30 min/day	-	10.7	3.3
	about 1 h/day	-	7.1	6.7
	about 2 h/day	20.00	14.3	6.7
	about 3 h/day	50.00	17.9	33.2
	about 4 h/day	30.00	25.0	16.7
	about 5 h/day	-	3.6	6.7
	about 6 h/day	-	-	3.3
	about 7 h or more/day	-	7.1	6.7
*p*	* 0.0876	** n/a	*** n/a	**** n/a
The number of hours spent playing games at the weekend—HBSC 5.2	none	13.3	21.5	16.7
	about 30 min/day	30.0	7.1	3.3
	about 1 h/day	16.7	-	6.7
	about 2 h/day	26.7	17.9	10.0
	about 3 h/day	13.3	7.1	20.0
	about 4 h/day	-	14.3	10.0
	about 5 h/day	-	10.7	10.0
	about 6 h/day	-	7.1	10.0
	about 7 h or more/day	-	14.3	13.3
*p*	* 0.0077	** 0.0028	*** 0.0021	**** 0.7355
The number of hours spent in front of a computer screen at the weekend—HBSC 6.2	none	-	10.7	-
	about 30 min/day	-	7.1	3.3
	about 1 h/day	43.3	3.6	3.3
	about 2 h/day	56.7	25.0	23.3
	about 3 h/day	-	17.9	16.7
	about 4 h/day	-	17.9	10.0
	about 5 h/day	-	7.1	16.7
	about 6 h/day	-	-	6.7
	about 7 h or more/day	-	10.7	20.0
*p*	* <0.0001	** <0.0001	*** <0.0001	**** 0.4277

Chi square test: * all groups, ** Group I versus Group III, *** Group I versus Group II, **** Group II versus Group III.

**Table 6:** Self-assessment of physical and mental health (KIDSCREEN). The percentage of respondents providing a given answer [%].

Question/Variable	Answer	Group I	Group II	Group III
Feeling fit and well—KID 1	not at all	40.00	3.6	-
	slightly	60.00	7.1	6.7
	moderately	-	53.6	10.0
	very	-	14.3	53.3
	extremely	-	21.4	30.0
*p*	* <0.0001	** <0.0001	*** <0.0001	**** 0.0022
Feeling of strength and energy—KID 2	never	23.3	-	-
	seldom	76.7	14.3	6.7
	quite often	-	46.4	23.3
	very often	-	14.3	26.7
	always	-	25.0	43.3
*p*	* <0.0001	** <0.0001	*** <0.0001	**** 0.1364
Feeling of sadness—KID 3	never	-	35.7	30.0
	seldom	-	46.4	56.7
	quite often	40.00	17.9	13.3
	very often	60.00	-	-
	always	-	-	-
*p*	* <0.0001	** <0.0001	*** <0.0001	**** 0.7302
Feeling of loneliness—KID 4	never	26.7	-	53.4
	seldom	50.0	50.0	40.0
	quite often	23.3	39.3	3.3
	very often	-	10.7	-
	always	-	-	3.3
*p*	* <0.0001	** 0.0367	*** 0.0078	**** <0.0001

Chi square test: * all groups, ** Group I vs. Group III, *** Group I vs. Group II, **** Group II vs. Group III.

**Table 7:** The ability to set standards for yourself (the autonomy of the child). The percentage of respondents providing a given answer [%].

Question/Variable	Answer	Group I	Group II	Group III
Having enough time for yourself—KID 5	never	-	-	3.3
	seldom	43.3	7.1	6.7
	quite often	53.4	28.6	23.3
	very often	3.3	39.3	36.7
	always	-	25.0	30.0
*p*	* <0.0001	** <0.0001	*** <0.0001	**** 0.8699
Performing the things that you want to do—KID 6	never	3.3	3.6	3.3
	seldom	30.0	7.1	3.3
	quite often	63.4	17.9	26.7
	very often	3.3	50.0	40.0
	always	-	21.4	26.7
*p*	* <0.0001	** <0.0001	*** <0.0001	**** 0.8446

Chi square test: * all groups, ** Group I versus Group III, *** Group I versus Group II, **** Group II versus Group III.

**Table 8:** Relationships with parents and peers. The percentage of respondents providing a given answer [%].

Question/Variable	Answer	Group I	Group II	Group III
Being treated fairly by parents/Good relations with parents—KID 7	never	-	-	-
	seldom	-	-	-
	quite often	-	7.1	20.00
	very often	10.00	42.9	13.3
	always	90.00	50.00	66.7
*p*	* 0.0008	** 0.0008	*** 0.0032	**** 0.0302
Good relations with peers, fun—KID 8	never	40.00	3.6	6.7
	seldom	60.00	7.1	3.3
	quite often	-	14.3	13.3
	very often	-	53.6	40.0
	always	-	21.4	36.7
*p*	* <0.0001	** <0.0001	*** <0.0001	**** 0.6618

Chi square test: * all groups, ** Group I versus Group III, *** Group I versus Group II, **** Group II versus Group III.

**Table 9:** School-related environment, concentration, and attention. The percentage of the respondents providing a given answer [%].

Question/Variable	Answer	Group I	Group II	Group III
Positive feelings related to the school environment—KID 9	not at all	26.7	-	-
	slightly	63.3	3.6	3.3
	moderately	10.0	42.9	30.0
	very	-	39.2	40.0
	extremely	-	14.3	26.7
*p*	* <0.0001	** <0.0001	*** <0.0001	**** 0.6284
Ability to pay attention—KID 10	never	23.3	3.6	-
	seldom	76.7	7.1	10.0
	quite often	-	46.5	23.4
	very often	-	21.4	33.3
	always	-	21.4	33.3
*p*	* <0.0001	** <0.0001	*** <0.0001	**** 0.2938

Chi square test: * all groups, ** Group I versus Group III, *** Group I versus Group II, **** Group II versus Group III.
